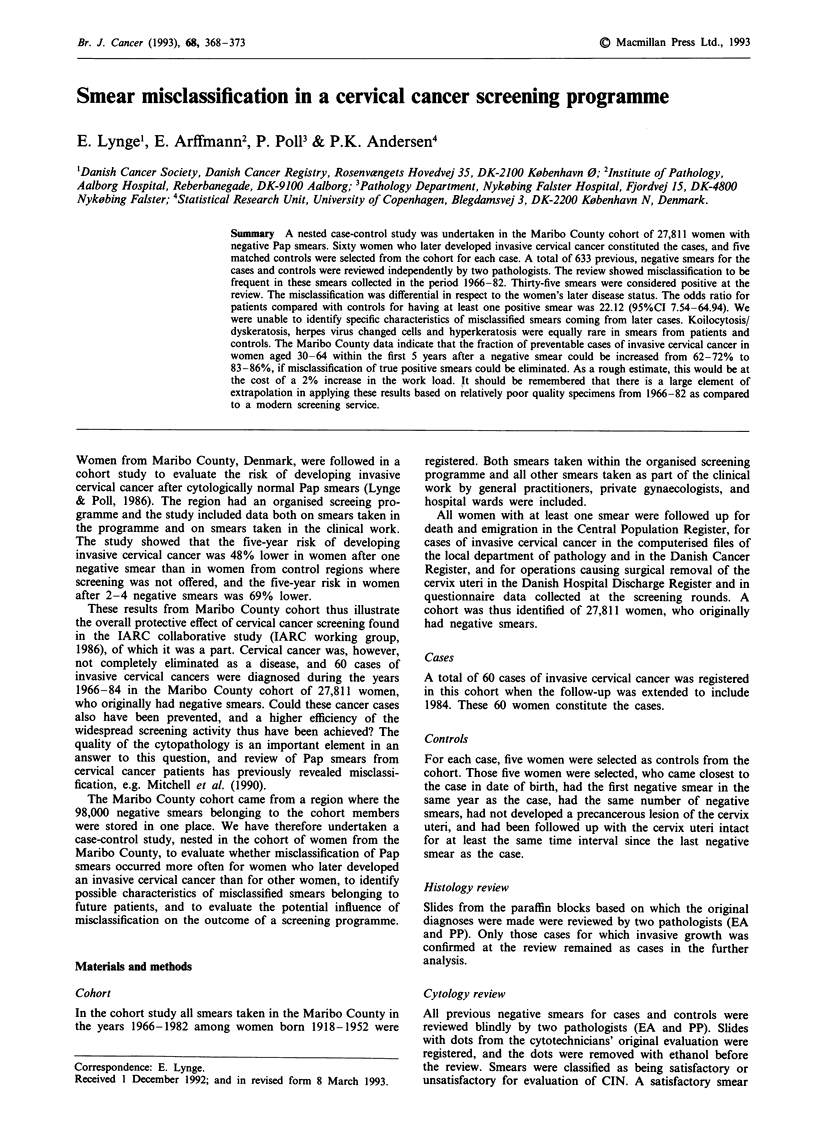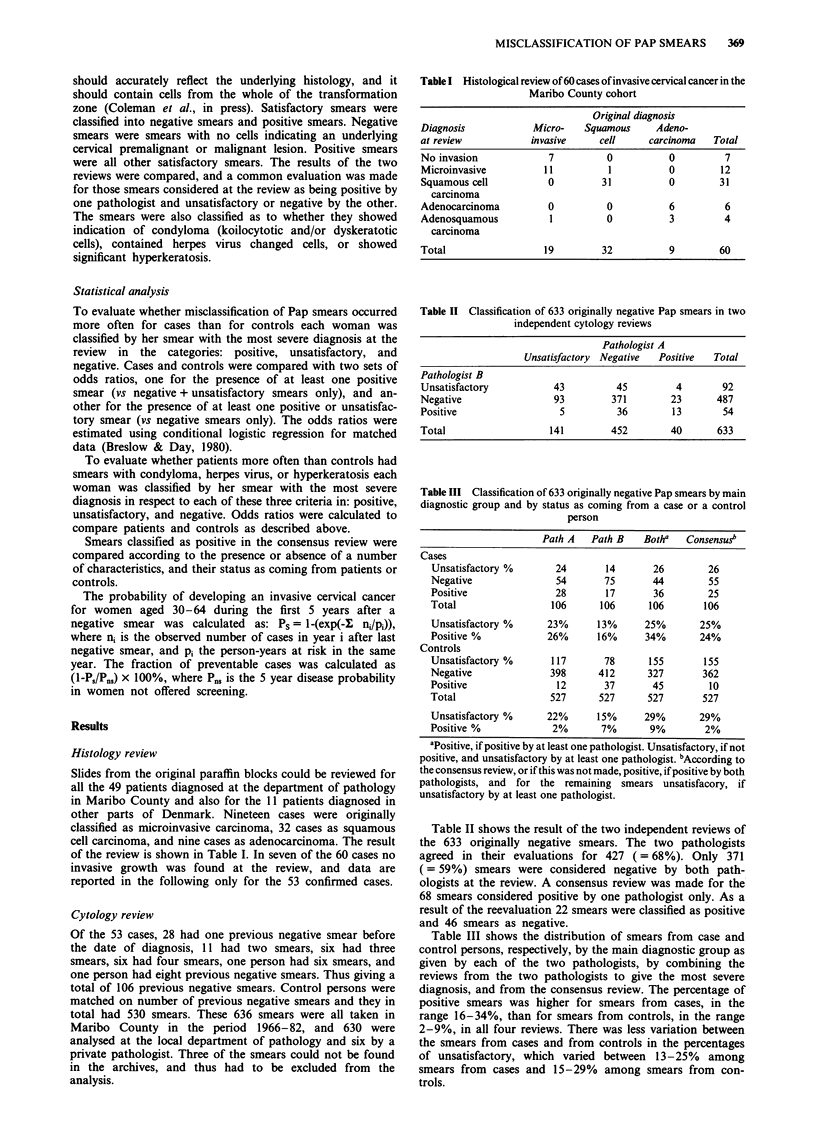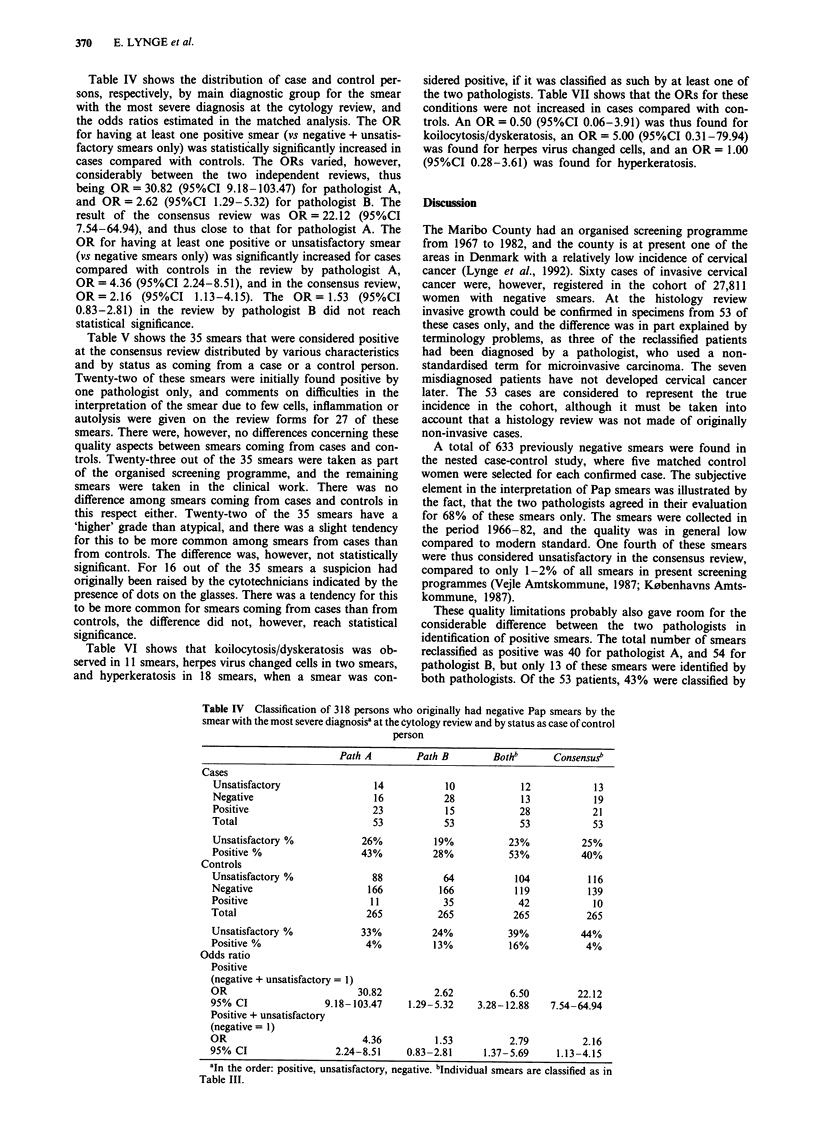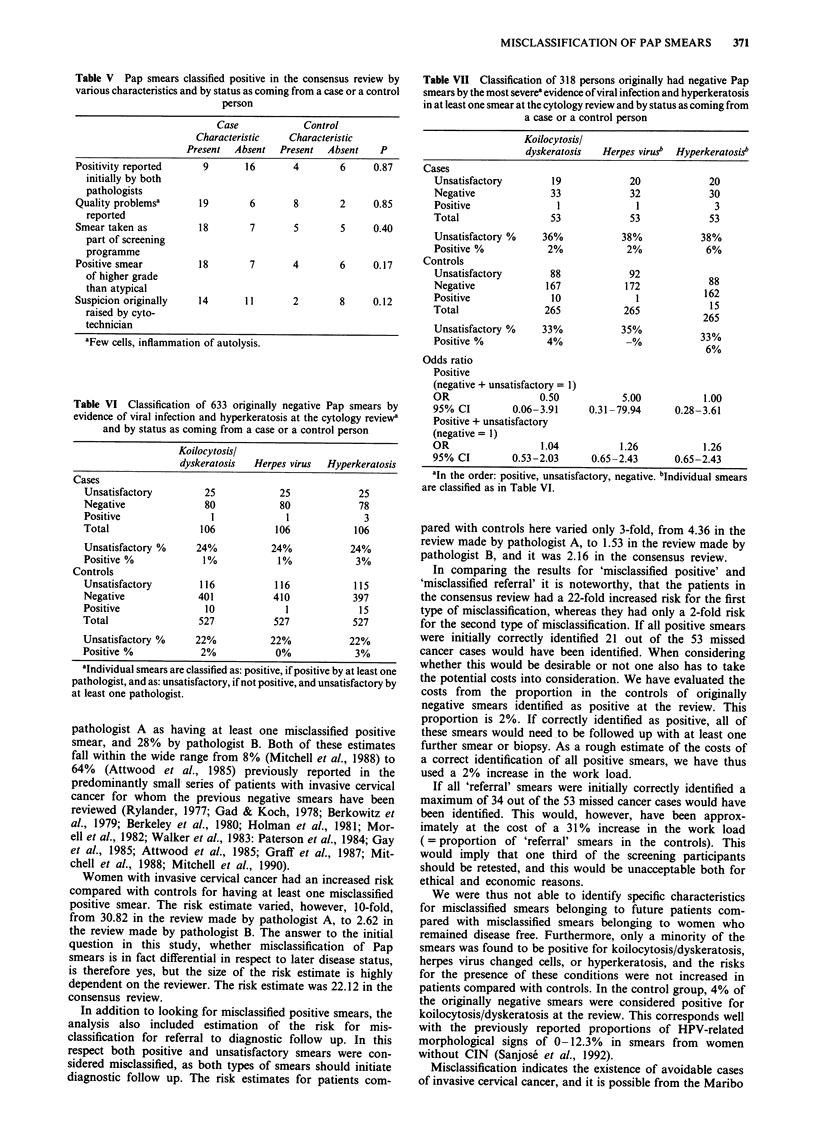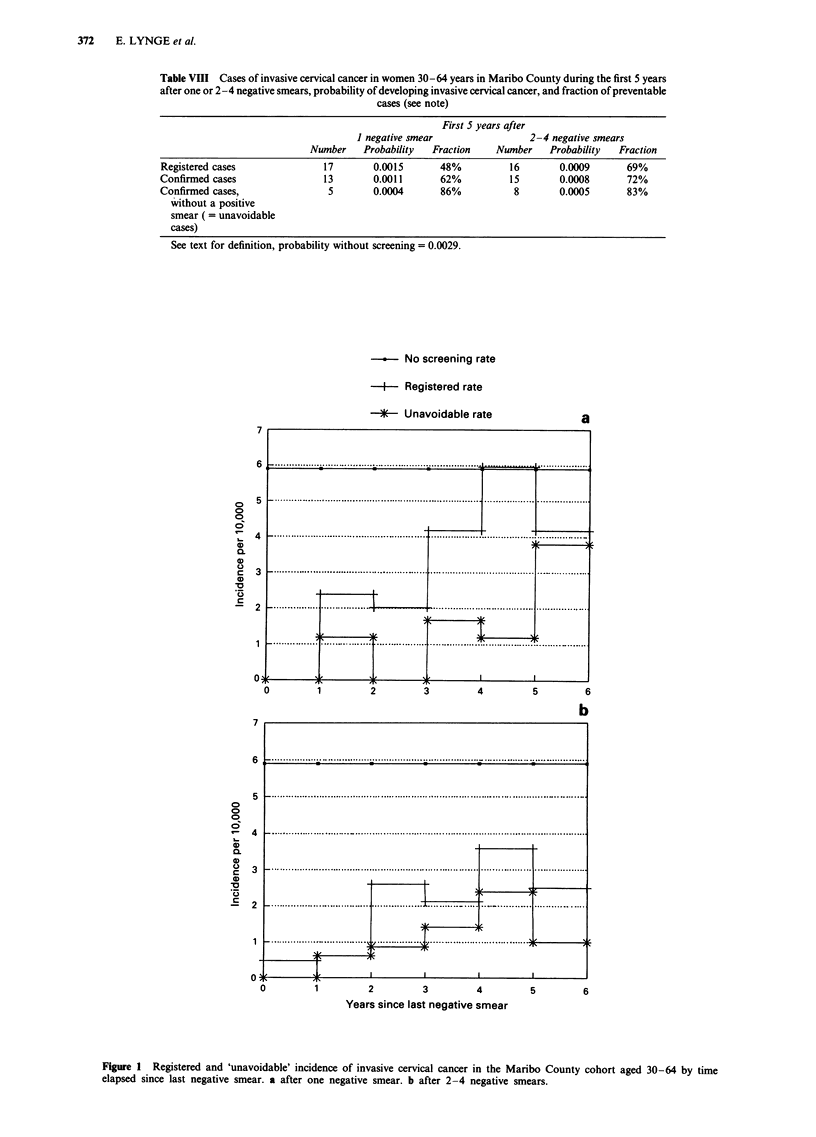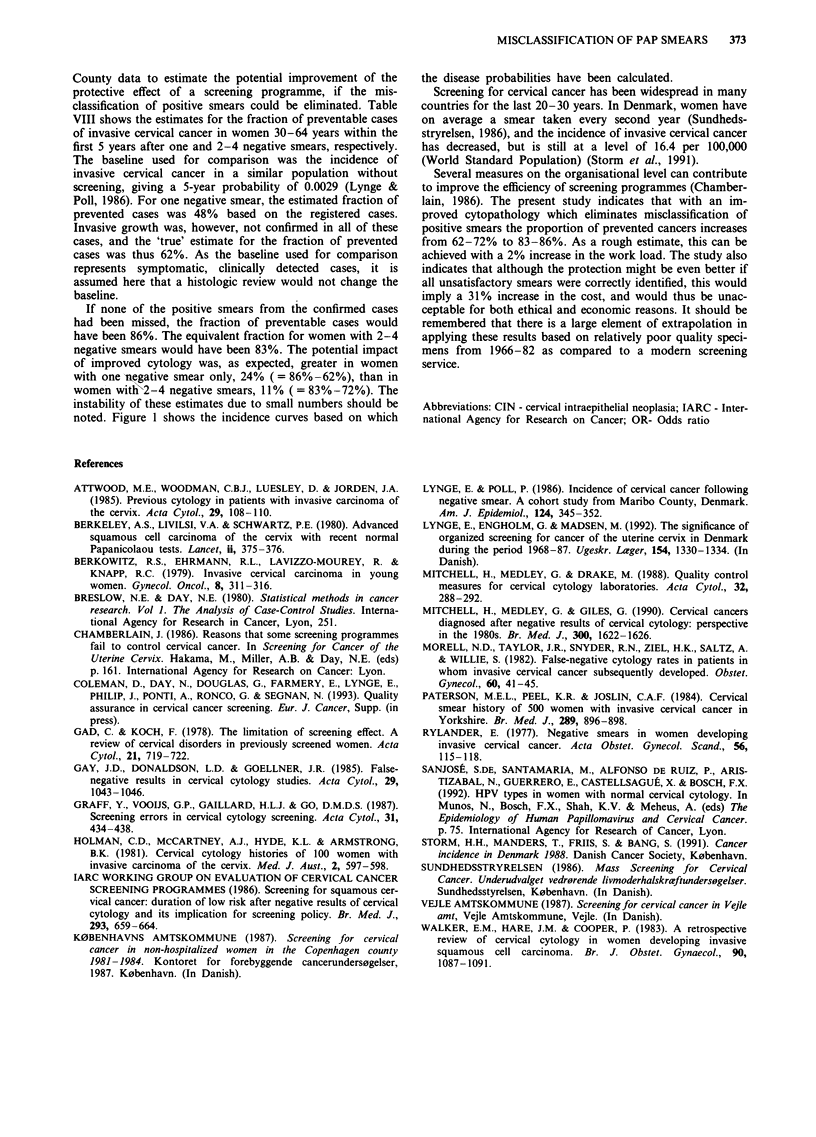# Smear misclassification in a cervical cancer screening programme.

**DOI:** 10.1038/bjc.1993.342

**Published:** 1993-08

**Authors:** E. Lynge, E. Arffmann, P. Poll, P. K. Anderson

**Affiliations:** Danish Cancer Society, Danish Cancer Registry, København.

## Abstract

A nested case-control study was undertaken in the Maribo County cohort of 27,811 women with negative Pap smears. Sixty women who later developed invasive cervical cancer constituted the cases, and five matched controls were selected from the cohort for each case. A total of 633 previous, negative smears for the cases and controls were reviewed independently by two pathologists. The review showed misclassification to be frequent in these smears collected in the period 1966-82. Thirty-five smears were considered positive at the review. The misclassification was differential in respect to the women's later disease status. The odds ratio for patients compared with controls for having at least one positive smear was 22.12 (95% CI 7.54-64.94). We were unable to identify specific characteristics of misclassified smears coming from later cases. Koilocytosis/dyskeratosis, herpes virus changed cells and hyperkeratosis were equally rare in smears from patients and controls. The Maribo County data indicate that the fraction of preventable cases of invasive cervical cancer in women aged 30-64 within the first 5 years after a negative smear could be increased from 62-72% to 83-86%, if misclassification of true positive smears could be eliminated. As a rough estimate, this would be at the cost of a 2% increase in the work load. It should be remembered that there is a large element of extrapolation in applying these results based on relatively poor quality specimens from 1966-82 as compared to a modern screening service.


Br. J. Cancer (1993), 68, 368-373 © Macmillan Press Ltd., 1993

### Smear misclassification in a cervical cancer screening programme

E. Lynge[1], E. Arffmann[2], P. Poll[3] & P.K. Andersen[4]

[1]*Danish Cancer Society, Danish Cancer Registry, Rosenvængets Hovedvej 35, DK-2100 København Ø;* [2]*Institute of Pathology, Aalborg Hospital, Reberbanegade, DK-9100 Aalborg;* [3]*Pathology Department, Nykøbing Falster Hospital, Fjordvej 15, DK-4800 Nykøbing Falster;* [4]*Statistical Research Unit, University of Copenhagen, Blegdamsvej 3, DK-2200 København N, Denmark.*

**Summary** A nested case-control study was undertaken in the Maribo County cohort of 27,811 women with negative Pap smears. Sixty women who later developed invasive cervical cancer constituted the cases, and five matched controls were selected from the cohort for each case. A total of 633 previous, negative smears for the cases and controls were reviewed independently by two pathologists. The review showed misclassification to be frequent in these smears collected in the period 1966-82. Thirty-five smears were considered positive at the review. The misclassification was differential in respect to the women's later disease status. The odds ratio for patients compared with controls for having at least one positive smear was 22.12 (95%CI 7.54-64.94). We were unable to identify specific characteristics of misclassified smears coming from later cases. Koilocytosis/dyskeratosis, herpes virus changed cells and hyperkeratosis were equally rare in smears from patients and controls. The Maribo County data indicate that the fraction of preventable cases of invasive cervical cancer in women aged 30-64 within the first 5 years after a negative smear could be increased from 62-72% to 83-86%, if misclassification of true positive smears could be eliminated. As a rough estimate, this would be at the cost of a 2% increase in the work load. It should be remembered that there is a large element of extrapolation in applying these results based on relatively poor quality specimens from 1966-82 as compared to a modern screening service.

Women from Maribo County, Denmark, were followed in a cohort study to evaluate the risk of developing invasive cervical cancer after cytologically normal Pap smears (Lynge & Poll, 1986). The region had an organised screeing programme and the study included data both on smears taken in the programme and on smears taken in the clinical work. The study showed that the five-year risk of developing invasive cervical cancer was 48% lower in women after one negative smear than in women from control regions where screening was not offered, and the five-year risk in women after 2-4 negative smears was 69% lower.

These results from Maribo County cohort thus illustrate the overall protective effect of cervical cancer screening found in the IARC collaborative study (IARC working group, 1986), of which it was a part. Cervical cancer was, however, not completely eliminated as a disease, and 60 cases of invasive cervical cancers were diagnosed during the years 1966-84 in the Maribo County cohort of 27,811 women, who originally had negative smears. Could these cancer cases also have been prevented, and a higher efficiency of the widespread screening activity thus have been achieved? The quality of the cytopathology is an important element in an answer to this question, and review of Pap smears from cervical cancer patients has previously revealed misclassification, e.g. Mitchell *et al.* (1990).

The Maribo County cohort came from a region where the 98,000 negative smears belonging to the cohort members were stored in one place. We have therefore undertaken a case-control study, nested in the cohort of women from the Maribo County, to evaluate whether misclassification of Pap smears occurred more often for women who later developed an invasive cervical cancer than for other women, to identify possible characteristics of misclassified smears belonging to future patients, and to evaluate the potential influence of misclassification on the outcome of a screening programme.

### Materials and methods

### *Cohort*

In the cohort study all smears taken in the Maribo County in the years 1966-1982 among women born 1918-1952 were registered. Both smears taken within the organised screening programme and all other smears taken as part of the clinical work by general practitioners, private gynaecologists, and hospital wards were included.

All women with at least one smear were followed up for death and emigration in the Central Population Register, for cases of invasive cervical cancer in the computerised files of the local department of pathology and in the Danish Cancer Register, and for operations causing surgical removal of the cervix uteri in the Danish Hospital Discharge Register and in questionnaire data collected at the screening rounds. A cohort was thus identified of 27,811 women, who originally had negative smears.

### *Cases*

A total of 60 cases of invasive cervical cancer was registered in this cohort when the follow-up was extended to include 1984. These 60 women constitute the cases.

### *Controls*

For each case, five women were selected as controls from the cohort. Those five women were selected, who came closest to the case in date of birth, had the first negative smear in the same year as the case, had the same number of negative smears, had not developed a precancerous lesion of the cervix uteri, and had been followed up with the cervix uteri intact for at least the same time interval since the last negative smear as the case.

### *Histology review*

Slides from the paraffin blocks based on which the original diagnoses were made were reviewed by two pathologists (EA and PP). Only those cases for which invasive growth was confirmed at the review remained as cases in the further analysis.

### *Cytology review*

All previous negative smears for cases and controls were reviewed blindly by two pathologists (EA and PP). Slides with dots from the cytotechnicians' original evaluation were registered, and the dots were removed with ethanol before the review. Smears were classified as being satisfactory or unsatisfactory for evaluation of CIN. A satisfactory smear

Correspondence: E. Lynge.
Received 1 December 1992; and in revised form 8 March 1993.

should accurately reflect the underlying histology, and it should contain cells from the whole of the transformation zone (Coleman *et al.*, in press). Satisfactory smears were classified into negative smears and positive smears. Negative smears were smears with no cells indicating an underlying cervical premalignant or malignant lesion. Positive smears were all other satisfactory smears. The results of the two reviews were compared, and a common evaluation was made for those smears considered at the review as being positive by one pathologist and unsatisfactory or negative by the other. The smears were also classified as to whether they showed indication of condyloma (koilocytotic and/or dyskeratotic cells), contained herpes virus changed cells, or showed significant hyperkeratosis.

### *Statistical analysis*

To evaluate whether misclassification of Pap smears occurred more often for cases than for controls each woman was classified by her smear with the most severe diagnosis at the review in the categories: positive, unsatisfactory, and negative. Cases and controls were compared with two sets of odds ratios, one for the presence of at least one positive smear (*vs* negative + unsatisfactory smears only), and another for the presence of at least one positive or unsatisfactory smear (*vs* negative smears only). The odds ratios were estimated using conditional logistic regression for matched data (Breslow & Day, 1980).

To evaluate whether patients more often than controls had smears with condyloma, herpes virus, or hyperkeratosis each woman was classified by her smear with the most severe diagnosis in respect to each of these three criteria in: positive, unsatisfactory, and negative. Odds ratios were calculated to compare patients and controls as described above.

Smears classified as positive in the consensus review were compared according to the presence or absence of a number of characteristics, and their status as coming from patients or controls.

The probability of developing an invasive cervical cancer for women aged 30–64 during the first 5 years after a negative smear was calculated as: $P_S = 1\text{-}(\exp(-\Sigma\ n_i/p_i))$, where $n_i$ is the observed number of cases in year i after last negative smear, and $p_i$ the person-years at risk in the same year. The fraction of preventable cases was calculated as $(1\text{-}P_s/P_{ns}) \times 100\%$, where $P_{ns}$ is the 5 year disease probability in women not offered screening.

### Results

### *Histology review*

Slides from the original paraffin blocks could be reviewed for all the 49 patients diagnosed at the department of pathology in Maribo County and also for the 11 patients diagnosed in other parts of Denmark. Nineteen cases were originally classified as microinvasive carcinoma, 32 cases as squamous cell carcinoma, and nine cases as adenocarcinoma. The result of the review is shown in Table I. In seven of the 60 cases no invasive growth was found at the review, and data are reported in the following only for the 53 confirmed cases.

### *Cytology review*

Of the 53 cases, 28 had one previous negative smear before the date of diagnosis, 11 had two smears, six had three smears, six had four smears, one person had six smears, and one person had eight previous negative smears. Thus giving a total of 106 previous negative smears. Control persons were matched on number of previous negative smears and they in total had 530 smears. These 636 smears were all taken in Maribo County in the period 1966–82, and 630 were analysed at the local department of pathology and six by a private pathologist. Three of the smears could not be found in the archives, and thus had to be excluded from the analysis.

**Table I** Histological review of 60 cases of invasive cervical cancer in the Maribo County cohort

| | *Original diagnosis* | | | |
|---|---|---|---|---|
| *Diagnosis at review* | *Micro-invasive* | *Squamous cell* | *Adeno-carcinoma* | *Total* |
| No invasion | 7 | 0 | 0 | 7 |
| Microinvasive | 11 | 1 | 0 | 12 |
| Squamous cell carcinoma | 0 | 31 | 0 | 31 |
| Adenocarcinoma | 0 | 0 | 6 | 6 |
| Adenosquamous carcinoma | 1 | 0 | 3 | 4 |
| Total | 19 | 32 | 9 | 60 |

**Table II** Classification of 633 originally negative Pap smears in two independent cytology reviews

| | *Pathologist A* | | | |
|---|---|---|---|---|
| | *Unsatisfactory* | *Negative* | *Positive* | *Total* |
| *Pathologist B* | | | | |
| Unsatisfactory | 43 | 45 | 4 | 92 |
| Negative | 93 | 371 | 23 | 487 |
| Positive | 5 | 36 | 13 | 54 |
| Total | 141 | 452 | 40 | 633 |

**Table III** Classification of 633 originally negative Pap smears by main diagnostic group and by status as coming from a case or a control person

| | *Path A* | *Path B* | *Both*[a] | *Consensus*[b] |
|---|---|---|---|---|
| Cases | | | | |
| Unsatisfactory % | 24 | 14 | 26 | 26 |
| Negative | 54 | 75 | 44 | 55 |
| Positive | 28 | 17 | 36 | 25 |
| Total | 106 | 106 | 106 | 106 |
| Unsatisfactory % | 23% | 13% | 25% | 25% |
| Positive % | 26% | 16% | 34% | 24% |
| Controls | | | | |
| Unsatisfactory % | 117 | 78 | 155 | 155 |
| Negative | 398 | 412 | 327 | 362 |
| Positive | 12 | 37 | 45 | 10 |
| Total | 527 | 527 | 527 | 527 |
| Unsatisfactory % | 22% | 15% | 29% | 29% |
| Positive % | 2% | 7% | 9% | 2% |

[a]Positive, if positive by at least one pathologist. Unsatisfactory, if not positive, and unsatisfactory by at least one pathologist. [b]According to the consensus review, or if this was not made, positive, if positive by both pathologists, and for the remaining smears unsatisfacory, if unsatisfactory by at least one pathologist.

Table II shows the result of the two independent reviews of the 633 originally negative smears. The two pathologists agreed in their evaluations for 427 ( = 68%). Only 371 ( = 59%) smears were considered negative by both pathologists at the review. A consensus review was made for the 68 smears considered positive by one pathologist only. As a result of the reevaluation 22 smears were classified as positive and 46 smears as negative.

Table III shows the distribution of smears from case and control persons, respectively, by the main diagnostic group as given by each of the two pathologists, by combining the reviews from the two pathologists to give the most severe diagnosis, and from the consensus review. The percentage of positive smears was higher for smears from cases, in the range 16–34%, than for smears from controls, in the range 2–9%, in all four reviews. There was less variation between the smears from cases and from controls in the percentages of unsatisfactory, which varied between 13–25% among smears from cases and 15–29% among smears from controls.

Table IV shows the distribution of case and control persons, respectively, by main diagnostic group for the smear with the most severe diagnosis at the cytology review, and the odds ratios estimated in the matched analysis. The OR for having at least one positive smear (*vs* negative + unsatisfactory smears only) was statistically significantly increased in cases compared with controls. The ORs varied, however, considerably between the two independent reviews, thus being OR = 30.82 (95%CI 9.18–103.47) for pathologist A, and OR = 2.62 (95%CI 1.29–5.32) for pathologist B. The result of the consensus review was OR = 22.12 (95%CI 7.54–64.94), and thus close to that for pathologist A. The OR for having at least one positive or unsatisfactory smear (*vs* negative smears only) was significantly increased for cases compared with controls in the review by pathologist A, OR = 4.36 (95%CI 2.24–8.51), and in the consensus review, OR = 2.16 (95%CI 1.13–4.15). The OR = 1.53 (95%CI 0.83–2.81) in the review by pathologist B did not reach statistical significance.

Table V shows the 35 smears that were considered positive at the consensus review distributed by various characteristics and by status as coming from a case or a control person. Twenty-two of these smears were initially found positive by one pathologist only, and comments on difficulties in the interpretation of the smear due to few cells, inflammation or autolysis were given on the review forms for 27 of these smears. There were, however, no differences concerning these quality aspects between smears coming from cases and controls. Twenty-three out of the 35 smears were taken as part of the organised screening programme, and the remaining smears were taken in the clinical work. There was no difference among smears coming from cases and controls in this respect either. Twenty-two of the 35 smears have a 'higher' grade than atypical, and there was a slight tendency for this to be more common among smears from cases than from controls. The difference was, however, not statistically significant. For 16 out of the 35 smears a suspicion had originally been raised by the cytotechnicians indicated by the presence of dots on the glasses. There was a tendency for this to be more common for smears coming from cases than from controls, the difference did not, however, reach statistical significance.

Table VI shows that koilocytosis/dyskeratosis was observed in 11 smears, herpes virus changed cells in two smears, and hyperkeratosis in 18 smears, when a smear was considered positive, if it was classified as such by at least one of the two pathologists. Table VII shows that the ORs for these conditions were not increased in cases compared with controls. An OR = 0.50 (95%CI 0.06–3.91) was thus found for koilocytosis/dyskeratosis, an OR = 5.00 (95%CI 0.31–79.94) was found for herpes virus changed cells, and an OR = 1.00 (95%CI 0.28–3.61) was found for hyperkeratosis.

### Discussion

The Maribo County had an organised screening programme from 1967 to 1982, and the county is at present one of the areas in Denmark with a relatively low incidence of cervical cancer (Lynge *et al.*, 1992). Sixty cases of invasive cervical cancer were, however, registered in the cohort of 27,811 women with negative smears. At the histology review invasive growth could be confirmed in specimens from 53 of these cases only, and the difference was in part explained by terminology problems, as three of the reclassified patients had been diagnosed by a pathologist, who used a non-standardised term for microinvasive carcinoma. The seven misdiagnosed patients have not developed cervical cancer later. The 53 cases are considered to represent the true incidence in the cohort, although it must be taken into account that a histology review was not made of originally non-invasive cases.

A total of 633 previously negative smears were found in the nested case-control study, where five matched control women were selected for each confirmed case. The subjective element in the interpretation of Pap smears was illustrated by the fact, that the two pathologists agreed in their evaluation for 68% of these smears only. The smears were collected in the period 1966–82, and the quality was in general low compared to modern standard. One fourth of these smears were thus considered unsatisfactory in the consensus review, compared to only 1–2% of all smears in present screening programmes (Vejle Amtskommune, 1987; Københavns Amtskommune, 1987).

These quality limitations probably also gave room for the considerable difference between the two pathologists in identification of positive smears. The total number of smears reclassified as positive was 40 for pathologist A, and 54 for pathologist B, but only 13 of these smears were identified by both pathologists. Of the 53 patients, 43% were classified by

**Table IV** Classification of 318 persons who originally had negative Pap smears by the smear with the most severe diagnosis[a] at the cytology review and by status as case of control person

| | *Path A* | *Path B* | *Both*[b] | *Consensus*[b] |
|---|---|---|---|---|
| Cases | | | | |
| Unsatisfactory | 14 | 10 | 12 | 13 |
| Negative | 16 | 28 | 13 | 19 |
| Positive | 23 | 15 | 28 | 21 |
| Total | 53 | 53 | 53 | 53 |
| Unsatisfactory % | 26% | 19% | 23% | 25% |
| Positive % | 43% | 28% | 53% | 40% |
| Controls | | | | |
| Unsatisfactory % | 88 | 64 | 104 | 116 |
| Negative | 166 | 166 | 119 | 139 |
| Positive | 11 | 35 | 42 | 10 |
| Total | 265 | 265 | 265 | 265 |
| Unsatisfactory % | 33% | 24% | 39% | 44% |
| Positive % | 4% | 13% | 16% | 4% |
| Odds ratio | | | | |
| Positive | | | | |
| (negative + unsatisfactory = 1) | | | | |
| OR | 30.82 | 2.62 | 6.50 | 22.12 |
| 95% CI | 9.18–103.47 | 1.29–5.32 | 3.28–12.88 | 7.54–64.94 |
| Positive + unsatisfactory | | | | |
| (negative = 1) | | | | |
| OR | 4.36 | 1.53 | 2.79 | 2.16 |
| 95% CI | 2.24–8.51 | 0.83–2.81 | 1.37–5.69 | 1.13–4.15 |

[a]In the order: positive, unsatisfactory, negative. [b]Individual smears are classified as in Table III.

**Table V** Pap smears classified positive in the consensus review by various characteristics and by status as coming from a case or a control person

| | *Case Characteristic* | | *Control Characteristic* | | |
|---|---|---|---|---|---|
| | *Present* | *Absent* | *Present* | *Absent* | *P* |
| Positivity reported initially by both pathologists | 9 | 16 | 4 | 6 | 0.87 |
| Quality problems[a] reported | 19 | 6 | 8 | 2 | 0.85 |
| Smear taken as part of screening programme | 18 | 7 | 5 | 5 | 0.40 |
| Positive smear of higher grade than atypical | 18 | 7 | 4 | 6 | 0.17 |
| Suspicion originally raised by cyto-technician | 14 | 11 | 2 | 8 | 0.12 |

[a]Few cells, inflammation of autolysis.

**Table VI** Classification of 633 originally negative Pap smears by evidence of viral infection and hyperkeratosis at the cytology review[a] and by status as coming from a case or a control person

| | *Koilocytosis/ dyskeratosis* | *Herpes virus* | *Hyperkeratosis* |
|---|---|---|---|
| Cases | | | |
| Unsatisfactory | 25 | 25 | 25 |
| Negative | 80 | 80 | 78 |
| Positive | 1 | 1 | 3 |
| Total | 106 | 106 | 106 |
| Unsatisfactory % | 24% | 24% | 24% |
| Positive % | 1% | 1% | 3% |
| Controls | | | |
| Unsatisfactory | 116 | 116 | 115 |
| Negative | 401 | 410 | 397 |
| Positive | 10 | 1 | 15 |
| Total | 527 | 527 | 527 |
| Unsatisfactory % | 22% | 22% | 22% |
| Positive % | 2% | 0% | 3% |

[a]Individual smears are classified as: positive, if positive by at least one pathologist, and as: unsatisfactory, if not positive, and unsatisfactory by at least one pathologist.

**Table VII** Classification of 318 persons originally had negative Pap smears by the most severe[a] evidence of viral infection and hyperkeratosis in at least one smear at the cytology review and by status as coming from a case or a control person

| | *Koilocytosis/ dyskeratosis* | *Herpes virus[b]* | *Hyperkeratosis[b]* |
|---|---|---|---|
| Cases | | | |
| Unsatisfactory | 19 | 20 | 20 |
| Negative | 33 | 32 | 30 |
| Positive | 1 | 1 | 3 |
| Total | 53 | 53 | 53 |
| Unsatisfactory % | 36% | 38% | 38% |
| Positive % | 2% | 2% | 6% |
| Controls | | | |
| Unsatisfactory | 88 | 92 | 88 |
| Negative | 167 | 172 | 162 |
| Positive | 10 | 1 | 15 |
| Total | 265 | 265 | 265 |
| Unsatisfactory % | 33% | 35% | 33% |
| Positive % | 4% | –% | 6% |
| Odds ratio | | | |
| Positive (negative + unsatisfactory = 1) | | | |
| OR | 0.50 | 5.00 | 1.00 |
| 95% CI | 0.06–3.91 | 0.31–79.94 | 0.28–3.61 |
| Positive + unsatisfactory (negative = 1) | | | |
| OR | 1.04 | 1.26 | 1.26 |
| 95% CI | 0.53–2.03 | 0.65–2.43 | 0.65–2.43 |

[a]In the order: positive, unsatisfactory, negative. [b]Individual smears are classified as in Table VI.

pathologist A as having at least one misclassified positive smear, and 28% by pathologist B. Both of these estimates fall within the wide range from 8% (Mitchell *et al.*, 1988) to 64% (Attwood *et al.*, 1985) previously reported in the predominantly small series of patients with invasive cervical cancer for whom the previous negative smears have been reviewed (Rylander, 1977; Gad & Koch, 1978; Berkowitz *et al.*, 1979; Berkeley *et al.*, 1980; Holman *et al.*, 1981; Morell *et al.*, 1982; Walker *et al.*, 1983: Paterson *et al.*, 1984; Gay *et al.*, 1985; Attwood *et al.*, 1985; Graff *et al.*, 1987; Mitchell *et al.*, 1988; Mitchell *et al.*, 1990).

Women with invasive cervical cancer had an increased risk compared with controls for having at least one misclassified positive smear. The risk estimate varied, however, 10-fold, from 30.82 in the review made by pathologist A, to 2.62 in the review made by pathologist B. The answer to the initial question in this study, whether misclassification of Pap smears is in fact differential in respect to later disease status, is therefore yes, but the size of the risk estimate is highly dependent on the reviewer. The risk estimate was 22.12 in the consensus review.

In addition to looking for misclassified positive smears, the analysis also included estimation of the risk for misclassification for referral to diagnostic follow up. In this respect both positive and unsatisfactory smears were considered misclassified, as both types of smears should initiate diagnostic follow up. The risk estimates for patients compared with controls here varied only 3-fold, from 4.36 in the review made by pathologist A, to 1.53 in the review made by pathologist B, and it was 2.16 in the consensus review.

In comparing the results for 'misclassified positive' and 'misclassified referral' it is noteworthy, that the patients in the consensus review had a 22-fold increased risk for the first type of misclassification, whereas they had only a 2-fold risk for the second type of misclassification. If all positive smears were initially correctly identified 21 out of the 53 missed cancer cases would have been identified. When considering whether this would be desirable or not one also has to take the potential costs into consideration. We have evaluated the costs from the proportion in the controls of originally negative smears identified as positive at the review. This proportion is 2%. If correctly identified as positive, all of these smears would need to be followed up with at least one further smear or biopsy. As a rough estimate of the costs of a correct identification of all positive smears, we have thus used a 2% increase in the work load.

If all 'referral' smears were initially correctly identified a maximum of 34 out of the 53 missed cancer cases would have been identified. This would, however, have been approximately at the cost of a 31% increase in the work load ( = proportion of 'referral' smears in the controls). This would imply that one third of the screening participants should be retested, and this would be unacceptable both for ethical and economic reasons.

We were thus not able to identify specific characteristics for misclassified smears belonging to future patients compared with misclassified smears belonging to women who remained disease free. Furthermore, only a minority of the smears was found to be positive for koilocytosis/dyskeratosis, herpes virus changed cells, or hyperkeratosis, and the risks for the presence of these conditions were not increased in patients compared with controls. In the control group, 4% of the originally negative smears were considered positive for koilocytosis/dyskeratosis at the review. This corresponds well with the previously reported proportions of HPV-related morphological signs of 0–12.3% in smears from women without CIN (Sanjosé *et al.*, 1992).

Misclassification indicates the existence of avoidable cases of invasive cervical cancer, and it is possible from the Maribo

**Table VIII** Cases of invasive cervical cancer in women 30–64 years in Maribo County during the first 5 years after one or 2–4 negative smears, probability of developing invasive cervical cancer, and fraction of preventable cases (see note)

| | First 5 years after | | | | | |
|---|---|---|---|---|---|---|
| | *1 negative smear* | | | *2–4 negative smears* | | |
| | *Number* | *Probability* | *Fraction* | *Number* | *Probability* | *Fraction* |
| Registered cases | 17 | 0.0015 | 48% | 16 | 0.0009 | 69% |
| Confirmed cases | 13 | 0.0011 | 62% | 15 | 0.0008 | 72% |
| Confirmed cases, without a positive smear ( = unavoidable cases) | 5 | 0.0004 | 86% | 8 | 0.0005 | 83% |

See text for definition, probability without screening = 0.0029.

**Figure 1** Registered and 'unavoidable' incidence of invasive cervical cancer in the Maribo County cohort aged 30–64 by time elapsed since last negative smear. **a** after one negative smear. **b** after 2–4 negative smears.

County data to estimate the potential improvement of the protective effect of a screening programme, if the misclassification of positive smears could be eliminated. Table VIII shows the estimates for the fraction of preventable cases of invasive cervical cancer in women 30–64 years within the first 5 years after one and 2–4 negative smears, respectively. The baseline used for comparison was the incidence of invasive cervical cancer in a similar population without screening, giving a 5-year probability of 0.0029 (Lynge & Poll, 1986). For one negative smear, the estimated fraction of prevented cases was 48% based on the registered cases. Invasive growth was, however, not confirmed in all of these cases, and the 'true' estimate for the fraction of prevented cases was thus 62%. As the baseline used for comparison represents symptomatic, clinically detected cases, it is assumed here that a histologic review would not change the baseline.

If none of the positive smears from the confirmed cases had been missed, the fraction of preventable cases would have been 86%. The equivalent fraction for women with 2–4 negative smears would have been 83%. The potential impact of improved cytology was, as expected, greater in women with one negative smear only, 24% ( = 86%–62%), than in women with 2–4 negative smears, 11% ( = 83%–72%). The instability of these estimates due to small numbers should be noted. Figure 1 shows the incidence curves based on which the disease probabilities have been calculated.

Screening for cervical cancer has been widespread in many countries for the last 20–30 years. In Denmark, women have on average a smear taken every second year (Sundhedsstyrelsen, 1986), and the incidence of invasive cervical cancer has decreased, but is still at a level of 16.4 per 100,000 (World Standard Population) (Storm *et al.*, 1991).

Several measures on the organisational level can contribute to improve the efficiency of screening programmes (Chamberlain, 1986). The present study indicates that with an improved cytopathology which eliminates misclassification of positive smears the proportion of prevented cancers increases from 62–72% to 83–86%. As a rough estimate, this can be achieved with a 2% increase in the work load. The study also indicates that although the protection might be even better if all unsatisfactory smears were correctly identified, this would imply a 31% increase in the cost, and would thus be unacceptable for both ethical and economic reasons. It should be remembered that there is a large element of extrapolation in applying these results based on relatively poor quality specimens from 1966–82 as compared to a modern screening service.

Abbreviations: CIN - cervical intraepithelial neoplasia; IARC - International Agency for Research on Cancer; OR- Odds ratio

### References

ATTWOOD, M.E., WOODMAN, C.B.J., LUESLEY, D. & JORDEN, J.A. (1985). Previous cytology in patients with invasive carcinoma of the cervix. *Acta Cytol.*, **29**, 108–110.

BERKELEY, A.S., LIVILSI, V.A. & SCHWARTZ, P.E. (1980). Advanced squamous cell carcinoma of the cervix with recent normal Papanicolaou tests. *Lancet*, **ii**, 375–376.

BERKOWITZ, R.S., EHRMANN, R.L., LAVIZZO-MOUREY, R. & KNAPP, R.C. (1979). Invasive cervical carcinoma in young women. *Gynecol. Oncol.*, **8**, 311–316.

BRESLOW, N.E. & DAY, N.E. (1980). *Statistical methods in cancer research. Vol 1. The Analysis of Case-Control Studies*. International Agency for Research in Cancer, Lyon, 251.

CHAMBERLAIN, J. (1986). Reasons that some screening programmes fail to control cervical cancer. In *Screening for Cancer of the Uterine Cervix*. Hakama, M., Miller, A.B. & Day, N.E. (eds) p. 161. International Agency for Research on Cancer: Lyon.

COLEMAN, D., DAY, N., DOUGLAS, G., FARMERY, E., LYNGE, E., PHILIP, J., PONTI, A., RONCO, G. & SEGNAN, N. (1993). Quality assurance in cervical cancer screening. *Eur. J. Cancer*, Supp. (in press).

GAD, C. & KOCH, F. (1978). The limitation of screening effect. A review of cervical disorders in previously screened women. *Acta Cytol.*, **21**, 719–722.

GAY, J.D., DONALDSON, L.D. & GOELLNER, J.R. (1985). False-negative results in cervical cytology studies. *Acta Cytol.*, **29**, 1043–1046.

GRAFF, Y., VOOIJS, G.P., GAILLARD, H.L.J. & GO, D.M.D.S. (1987). Screening errors in cervical cytology screening. *Acta Cytol.*, **31**, 434–438.

HOLMAN, C.D., MCCARTNEY, A.J., HYDE, K.L. & ARMSTRONG, B.K. (1981). Cervical cytology histories of 100 women with invasive carcinoma of the cervix. *Med. J. Aust.*, **2**, 597–598.

IARC WORKING GROUP ON EVALUATION OF CERVICAL CANCER SCREENING PROGRAMMES (1986). Screening for squamous cervical cancer: duration of low risk after negative results of cervical cytology and its implication for screening policy. *Br. Med. J.*, **293**, 659–664.

KØBENHAVNS AMTSKOMMUNE (1987). *Screening for cervical cancer in non-hospitalized women in the Copenhagen county 1981–1984*. Kontoret for forebyggende cancerundersøgelser, 1987. København. (In Danish).

LYNGE, E. & POLL, P. (1986). Incidence of cervical cancer following negative smear. A cohort study from Maribo County, Denmark. *Am. J. Epidemiol.*, **124**, 345–352.

LYNGE, E., ENGHOLM, G. & MADSEN, M. (1992). The significance of organized screening for cancer of the uterine cervix in Denmark during the period 1968–87. *Ugeskr. Læger*, **154**, 1330–1334. (In Danish).

MITCHELL, H., MEDLEY, G. & DRAKE, M. (1988). Quality control measures for cervical cytology laboratories. *Acta Cytol.*, **32**, 288–292.

MITCHELL, H., MEDLEY, G. & GILES, G. (1990). Cervical cancers diagnosed after negative results of cervical cytology: perspective in the 1980s. *Br. Med. J.*, **300**, 1622–1626.

MORELL, N.D., TAYLOR, J.R., SNYDER, R.N., ZIEL, H.K., SALTZ, A. & WILLIE, S. (1982). False-negative cytology rates in patients in whom invasive cervical cancer subsequently developed. *Obstet. Gynecol.*, **60**, 41–45.

PATERSON, M.E.L., PEEL, K.R. & JOSLIN, C.A.F. (1984). Cervical smear history of 500 women with invasive cervical cancer in Yorkshire. *Br. Med. J.*, **289**, 896–898.

RYLANDER, E. (1977). Negative smears in women developing invasive cervical cancer. *Acta Obstet. Gynecol. Scand.*, **56**, 115–118.

SANJOSÉ, S.DE, SANTAMARIA, M., ALFONSO DE RUIZ, P., ARISTIZABAL, N., GUERRERO, E., CASTELLSAGUÉ, X. & BOSCH, F.X. (1992). HPV types in women with normal cervical cytology. In Munos, N., Bosch, F.X., Shah, K.V. & Meheus, A. (eds) *The Epidemiology of Human Papillomavirus and Cervical Cancer*. p. 75. International Agency for Research of Cancer, Lyon.

STORM, H.H., MANDERS, T., FRIIS, S. & BANG, S. (1991). *Cancer incidence in Denmark 1988*. Danish Cancer Society, København.

SUNDHEDSSTRYRELSEN (1986). *Mass Screening for Cervical Cancer. Underudvalget vedrørende livmoderhalskræftundersøgelser*. Sundhedsstyrelsen, København. (In Danish).

VEJLE AMTSKOMMUNE (1987). *Screening for cervical cancer in Vejle amt*, Vejle Amtskommune, Vejle. (In Danish).

WALKER, E.M., HARE, J.M. & COOPER, P. (1983). A retrospective review of cervical cytology in women developing invasive squamous cell carcinoma. *Br. J. Obstet. Gynaecol.*, **90**, 1087–1091.